# Integrated-photonics-based systems for polarization-gradient cooling of trapped ions

**DOI:** 10.1038/s41377-025-02094-4

**Published:** 2026-01-15

**Authors:** Sabrina M. Corsetti, Ashton Hattori, Ethan R. Clements, Felix W. Knollmann, Milica Notaros, Reuel Swint, Tal Sneh, Patrick T. Callahan, Gavin N. West, Dave Kharas, Thomas Mahony, Colin D. Bruzewicz, Cheryl Sorace-Agaskar, Robert McConnell, Isaac L. Chuang, John Chiaverini, Jelena Notaros

**Affiliations:** 1 Research Laboratory of Electronics, Massachusetts Institute of Technology, Cambridge, MA 02139 USA; 2 Lincoln Laboratory, Massachusetts Institute of Technology, Lexington, MA 02421 USA

**Keywords:** Integrated optics, Atom optics

## Abstract

Trapped ions are a promising modality for quantum systems, with demonstrated utility as the basis for quantum processors and optical clocks. However, traditional trapped-ion systems are implemented using complex free-space optical configurations, whose large size and susceptibility to vibrations and drift inhibit scaling to large numbers of qubits. In recent years, integrated-photonics-based systems have been demonstrated as an avenue to address the challenge of scaling trapped-ion systems while maintaining high fidelities. While these previous demonstrations have implemented both Doppler and resolved-sideband cooling of trapped ions, these cooling techniques are fundamentally limited in efficiency. In contrast, polarization-gradient cooling can enable faster and more power-efficient cooling and, therefore, improved computational efficiencies in trapped-ion systems. While free-space implementations of polarization-gradient cooling have demonstrated advantages over other cooling mechanisms, polarization-gradient cooling has never previously been implemented using integrated photonics. In this paper, we design and experimentally demonstrate key polarization-diverse integrated-photonics devices and utilize them to implement a variety of integrated-photonics-based polarization-gradient-cooling systems, culminating in the first experimental demonstration of polarization-gradient cooling of a trapped ion by an integrated-photonics-based system. By demonstrating polarization-gradient cooling using an integrated-photonics-based system and, in general, opening up the field of polarization-diverse integrated-photonics-based devices and systems for trapped ions, this work facilitates new capabilities for integrated-photonics-based trapped-ion platforms.

## Abstract (web summary)

We design and demonstrate a variety of integrated-photonics-based polarization-gradient-cooling systems, culminating in the first experimental demonstration of trapped-ion polarization-gradient cooling using integrated photonics, facilitating new capabilities for integrated-photonics-based trapped-ion platforms.

## Graphical abstract

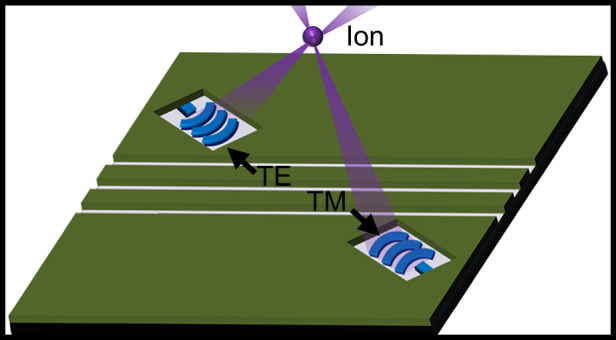

## Introduction

The intrinsic reproducibility, long coherence times, and strong interactions available in collections of trapped atomic ions have enabled a variety of quantum logic operations with fidelities sufficient for fault-tolerant quantum computing, including single- and two-qubit gates, qubit state preparation, and readout^1–4^. At the system level, trapped ions have been successfully implemented as the basis for quantum processors and high-accuracy optical clocks^5–11^. However, scaling trapped-ion systems to a large number of qubits while maintaining high fidelities remains a significant challenge for achieving practical, general, and portable quantum systems^4,12,13^.

Traditionally, trapped-ion systems have been implemented using complex free-space optical configurations, whose large size and susceptibility to vibrations and drift can limit the fidelity and addressability of ion arrays, inhibiting scaling to large numbers of qubits. Recently, integrated-photonics-based devices and systems have been demonstrated as an avenue to address these challenges by enabling photonic routing and optical-beam emission from systems that offer a scalable chip-based form factor and inherently-vibration-resilient optical addressing^12–15^. For example, foundational works have developed and utilized low-loss integrated-photonics platforms^16^ to enable trapped-ion operations ranging from photoionization and cooling^12^ to two-qubit operations^13^. More recent demonstrations have further advanced the field, demonstrating steps towards scaled multi-zone operation of integrated-photonics-based ion-trap chips^14,15^.

While these previous demonstrations have utilized integrated-photonics-based beam delivery to implement both Doppler and resolved-sideband cooling, these cooling techniques are respectively limited by a higher final kinetic energy (the Doppler cooling limit)^17,18^ and the inherent slowness of addressing individual motional sidebands^19,20^. Overcoming these limitations is of particular interest for improving trapped-ion computational efficiencies, as inefficient ground-state cooling can comprise a significant portion of a trapped-ion system’s computational time^21–23^. Compared to Doppler and resolved-sideband cooling, alternative cooling mechanisms provide useful tradeoffs in rate and final temperature to enable faster and more power-efficient cooling and, therefore, improved computational efficiencies for trapped-ion systems.

Two such higher-efficiency cooling mechanisms are electromagnetically-induced-transparency cooling and polarization-gradient cooling. While electromagnetically-induced-transparency cooling offers rapid ground-state cooling, this cooling mechanism becomes slow when applied to low-frequency motional modes, which are of particular interest for large ion chains confined in linear Paul traps^24^. Alternatively, polarization-gradient cooling offers the potential for parallel cooling of multiple motional modes spanning a broader frequency range to below the Doppler limit, making it a promising technique for efficiently transitioning between Doppler and ground-state cooling techniques^25^. Prior demonstrations of polarization-gradient cooling have successfully cooled chains of up to 51 ions to sub-Doppler temperatures over multiple motional modes simultaneously, demonstrating the practicality and benefit of implementing polarization-gradient cooling within multi-ion systems^24,26–28^.

However, all existing demonstrations of polarization-gradient cooling to date have been implemented using free-space beam delivery; polarization-gradient cooling has never previously been implemented using integrated photonics. This is primarily due to the stringent beam-delivery requirements imposed by polarization-gradient cooling. While both Doppler and resolved-sideband cooling can be performed using only a single linearly-polarized beam in some atomic species, polarization-gradient cooling relies on the interaction between an atom and a polarization gradient generated by the interference between either two circularly-polarized beams or two beams with orthogonal linear polarizations^25^. To enable such configurations using integrated photonics, multiple emitters must be arranged to simultaneously deliver beams to a target ion location. Furthermore, some configurations require the emission of beams with different polarizations. These configurations necessitate the co-optimization of multiple polarization-diverse emitters for equal-intensity beam formation. Advances have been made in recent years targeting polarization-diverse integrated-photonics devices for qubit interactions^29–31^, and multi-beam trapped-ion functionalities have been demonstrated using integrated-photonics-based trap chips, such as simultaneous Doppler cooling and repumping^12^; however, all previous demonstrations of cooling performed by integrated-photonics-based trapped-ion systems have been limited to emission of single transverse-electric-polarized (TE-polarized) beams^12–15^, thus inhibiting the implementation of polarization-gradient cooling.

In this paper, we design and experimentally demonstrate key polarization-diverse integrated-photonics devices and utilize them to implement a variety of integrated-photonics-based polarization-gradient-cooling systems, culminating in the first integrated-photonics-based demonstration of polarization-gradient cooling of a trapped ion. First, we discuss the mechanisms underlying polarization-gradient cooling and introduce several integrated-photonics-based architectures capable of performing polarization-gradient cooling. Second, we design and experimentally demonstrate the key integrated-photonics routing and grating-emitter devices required to implement these architectures. Third, we incorporate these devices into, and successfully experimentally demonstrate, multiple integrated-photonics-based polarization-gradient-cooling systems capable of forming either traveling-wave or phase-stable polarization gradients. Finally, we implement one of the phase-stable systems within an integrated-photonics-based ion-trap chip and use it to demonstrate the first polarization-gradient cooling of a trapped ion using an integrated-photonics-based system. (A broader characterization of the ion dynamics resulting from this phase-stable polarization gradient is presented in detail in our companion paper^32^.) By demonstrating polarization-gradient cooling using an integrated-photonics-based system and, in general, opening up the field of polarization-diverse integrated-photonics-based devices and systems for trapped ions, this work facilitates new capabilities for integrated-photonics-based trapped-ion platforms.

## Results

### Conceptual overview of integrated-photonics-based architectures for polarization-gradient cooling

**Fig. 1:**
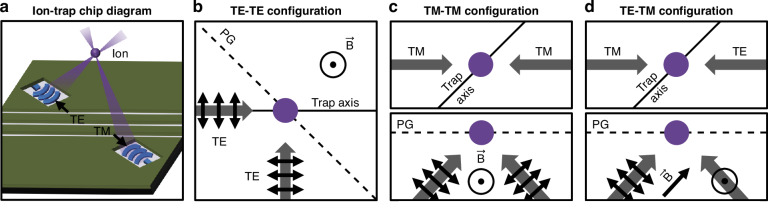
Integrated-photonics-based polarization-gradient-cooling concept. **a** Conceptual diagram of a planar electronic-photonic trap chip containing integrated-photonics gratings emitting optical beams upward towards a common ion location through surface-electrode windows (not to scale). **b**–**d** Conceptual diagrams of the polarization-gradient-cooling configurations explored in this work, depicting the beam (gray arrows), beam-polarization (black arrows), polarization-gradient (PG), and magnetic-field ($\vec{B}$) orientations. **b** Top-view conceptual diagram of a TE-TE polarization-gradient-cooling configuration. **c** Top-view (top) and side-view (bottom) conceptual diagrams of a TM-TM polarization-gradient-cooling configuration. **d** Top-view (top) and side-view (bottom) conceptual diagrams of a TE-TM polarization-gradient-cooling configuration

**Fig. 2:**
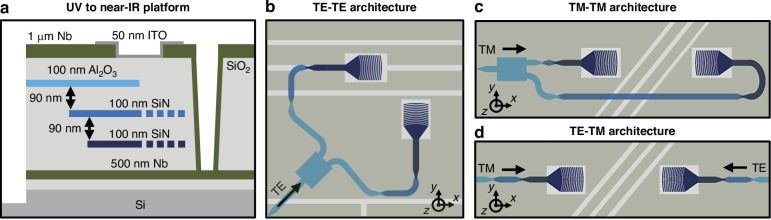
Integrated-photonics-based polarization-gradient-cooling architecture implementation. **a** Simplified schematic (not to scale) of the low-loss UV-to-near-IR 200-mm wafer-scale platform^16^ used to fabricate the electronic-photonic trap chip utilized in this work, consisting of three photonic waveguiding layers in Al_2_O_3_ and SiN, an Nb surface-electrode layer, an Nb ground plane, and an ITO dielectric-shielding layer. Top-view schematics (not to scale) of the (**b**) TE-TE, (**c**) TM-TM, and (**d**) TE-TM architectures designed and demonstrated in this work, illustrating the integrated-photonics devices implemented in Al_2_O_3_, single-layer SiN, and dual-layer SiN, as well as the alignment of the Nb surface electrodes with respect to the dual-layer SiN grating emitters

Integrated-photonics-based ion-trap chips build upon traditional surface-electrode-based trap chips by integrating both photonics layers and surface electrodes into a single planar electronic-photonic trap chip^12–15^. The surface electrodes allow for confinement and routing of ions within radio-frequency traps formed over the trap-chip surface^33^. Integrated-photonics layers underneath the electrodes allow for routing of light towards integrated grating couplers that emit optical beams towards the trapped ions through gaps in the surface electrodes, as conceptually depicted in Figs. 1a and 2a.

In prior work^12^, individual integrated gratings emitting TE-polarized light were used analogously to linearly-polarized free-space beams to demonstrate Doppler and resolved-sideband cooling of a trapped ^88^Sr^+^ ion using an integrated-photonics-based system. First, integrated-photonics-based Doppler cooling along the ^88^Sr^+^ ${\rm S{_{1/2}}} \rightarrow{\rm P{_{1/2}}}$ transition was enabled using a 422-nm-wavelength beam. The further implementation of integrated-photonics-based resolved-sideband cooling by driving the ${\rm S{_{1/2}}} \rightarrow{\rm D{_{5/2}}}$ transition using a 674-nm beam, coupled with pumping along the ${\rm D{_{5/2}}} \rightarrow{\rm P{_{3/2}}}$ transition using a 1033-nm beam, enabled cooling of the average axial-mode motional-state occupation to $\bar{n} < 1$. In this work, we extend beyond these previously demonstrated cooling techniques by enabling polarization-gradient cooling using polarization-diverse integrated-photonics-based devices and systems, paving a path towards enhanced trapped-ion state-preparation and computational efficiencies^21^.

As introduced above, polarization-gradient cooling is enabled by the interaction of multiple beams with either orthogonal linear polarizations (the lin $\perp$ lin configuration) or opposite-handed circular polarizations (the ${\sigma }^{+}$- ${\sigma }^{-}$ configuration)^25^. In this work, we focus on the lin $\perp$ lin configuration. In the ideal case, this configuration is implemented using two counterpropagating beams with wavelength $\lambda$. The orthogonal polarizations of the beams create a polarization gradient of varying ellipticity (between circular and linear polarization) along the propagation axis of the beams. To summarize the theory underlying the cooling mechanism performed by this ideal gradient (as first introduced in^25^), we consider an ion with ground-state Zeeman sublevels *g*_*±*1/2_ subject to light shifts induced by the polarization gradient. In regions of pure ${\sigma }^{-}$ circular polarization, the *g*_1/2_ ground state is strongly coupled to the light field and experiences a large light shift, while the *g*_-1/2_ state remains unaffected. The converse relationship holds for ${\sigma }^{+}$ polarization. As a result, the light-shifted energy levels of *g*_±1/*2*_ obtain a periodic spatial dependence. By choosing the laser detuning such that the ion is optically pumped to the lower-energy Zeeman sublevel during its motion, we enact motional-state cooling by means of periodic stimulated loss of kinetic energy^25^.

In practice, many beam and magnetic-field orientations are conducive to lin $\perp$ lin polarization-gradient cooling. Ideally, the polarization vectors and applied magnetic fields comprising any given cooling configuration will be mutually orthogonal, resulting in the formation of a polarization gradient with alternating ${\sigma }^{+}$- ${\sigma }^{-}$ polarization at the intersection of the beams. Figure 1b-d depicts three example polarization-gradient-cooling configurations explored in this work that are compatible with being emitted by a planar trap chip. Since it is challenging to emit counterpropagating beams from a planar trap chip that intersect at an ion location, these configurations consist of orthogonally-polarized beams emitted by the trap chip with a near-45° angle from the vertical such that the beams intersect at a target ion location over the trap-chip surface, as conceptually depicted in Fig. 1a. For each of the configurations in this work, we aim to enable simultaneous cooling of all three modes of a trapped ion’s motion by offsetting the polarization gradient from the trap axis by 45° in the plane of the trap chip and applying appropriate static voltages to rotate the combined radio-frequency and static potential such that the polarization gradient has a component along all three principal trap axes.

To physically implement each of these three beam configurations, we integrate two grating emitters into an electronic-photonic trap site, positioned equidistant from a target ion location. Each integrated grating emits a focused 422-nm-wavelength beam at a near-45° angle from the vertical towards the ion located 50 µm over the surface of the chip. These architectures, depicted in Fig. 2b–d, include two TE-polarization-emitting gratings placed orthogonal to each other (TE-TE), two transverse-magnetic-polarization-emitting (TM-polarization-emitting) gratings oriented opposite each other (TM-TM), and a TE- and a TM-polarization-emitting grating placed opposite each other (TE-TM).

These varying architectures allow for flexibility in the orientation of the gratings. As an example, this is especially useful for trap sites that require many concentric integrated grating emitters to enable multiple ion functionalities at the same site^12^. Furthermore, as each architecture corresponds to a different magnetic-field orientation relative to the chip plane, the varying architectures can each provide advantages for implementing additional functionalities using integrated photonics. For example, in the particular case of the TE-TM architecture, the corresponding magnetic-field orientation falls at an off-axis angle with respect to the chip plane; therefore, in the TE-TM case, it is comparatively simple to implement additional functionalities requiring the emission of circular polarization parallel to the magnetic field, such as selective optical pumping for state preparation^34^, since an integrated grating capable of emitting a single circularly-polarized beam^29^ can be designed and oriented such that the emitted beam is parallel to the magnetic field.

### Design of polarization-diverse grating-emitter pairs

As a central component of these polarization-gradient-cooling architectures, we must design integrated grating emitters capable of emitting intensity-matched beams of diverse polarizations from a planar trap chip^35^. In this section, we design a pair of integrated TE- and TM-emitting gratings with an operating wavelength of 422 nm, corresponding to the ^88^Sr^+^ ${\rm S{_{1/2}}} \rightarrow{\rm P{_{1/2}}}$ transition.

**Fig. 3:**
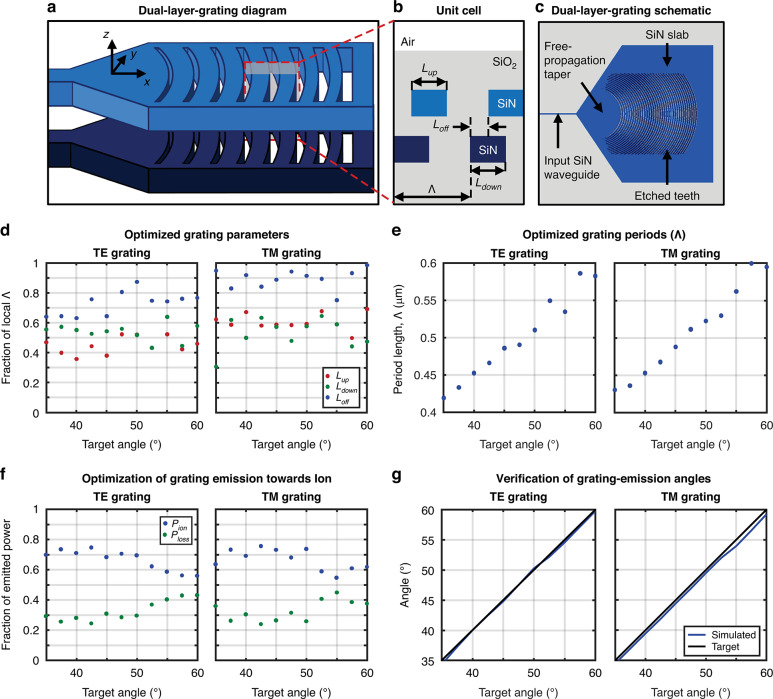
Polarization-diverse integrated-photonics grating-emitter design process. **a** Simplified diagram (not to scale) of our dual-layer SiN integrated-photonics grating emitters, demonstrating apodization and curvature of the grating teeth for focusing-beam emission. **b** Cross-sectional view (not to scale) of two unit cells of the grating depicted in (**a**), highlighting our optimization parameters: period length ($\varLambda$), upper-tooth length (*L*_*up*_), lower-tooth length (*L*_*down*_), and offset between layers in the *x* dimension (*L*_*off*_). **c** Top-view schematic of a designed TE grating; light enters from the dual-layer SiN waveguide on the left, transitions through a free-propagation taper region, and reaches the grating region, which consists of SiO_2_ teeth (gray) etched into the dual-layer SiN (blue). TE- and TM-grating optimization-routine results, depicting the optimized values of (**d**) *L*_*up*_, *L*_*down*_, and *L*_*off*_ (as a fraction of $\varLambda$) and (**e**) $\varLambda$ for each target angle of emission; (**f**) the maximization of optical power emitted towards a desired ion location as a fraction of the total emitted optical power (*P*_*ion*_) and minimization of optical power emitted in all other directions as a fraction of the total emitted optical power (*P*_*loss*_) for each target emission angle; and (**g**) the simulated resulting emission angles versus the desired target emission angles

To maximize the percentage of light emitted towards a target ion location 50 μm above the surface of the chip, we design the gratings to emit focused beams unidirectionally upwards. To achieve this, we implement the gratings using a bilayer, apodized, and curved geometry. The bilayer structure of the gratings enables unidirectional emission upwards, and the grating apodization and curvature enable focused beam emission. As depicted in Fig. 2a, the gratings are composed of two 100-nm-thick layers of silicon nitride (SiN) separated by 90 nm of silicon dioxide (SiO_2_). Similar to^36^, we apodize the periods and lengths of each layer’s grating teeth such that the angle of emission varies along the length of the grating to enable focusing in the propagation dimension (*x*), as depicted in Fig. 3a. In addition, similar to^37^, we utilize the bilayer grating structure to enable unidirectional emission upwards by offsetting the teeth in each layer from each other in the propagation dimension (*x*), as depicted in Fig. 3b. Finally, we curve the grating teeth to enable focusing in the transverse dimension (*y*) of the grating, as depicted in Fig. 3c. The gratings are fed by a 10-μm-long large-angle free-propagation taper such that the input fundamental waveguide mode propagating in the bilayer silicon-nitride waveguide freely expands up to the full width of the grating.

To start the design process, we first define the physical features and constraints of our gratings: the fabrication stack, minimum fabricable feature size, grating aperture size, target ion location, operating wavelength, and input polarization (TE or TM). Then, using these values as guidelines, we determine a set of optimal grating parameters (upper-layer tooth length, lower-layer tooth length, layer offset in the *x* dimension, and period length as labeled in Fig. 3b) for each period within the grating. Given that all four of these parameters must be uniquely optimized for each of the approximately forty periods within the grating, this multivariable optimization would be complex and computationally demanding to perform using traditional design techniques^38^. Accordingly, we instead optimize these parameters numerically using a particle-swarm algorithm, a common choice of algorithm for multivariable grating optimization^39,40^_._

To perform this optimization, we first utilize the particle-swarm algorithm to generate a dataset that maps a discrete number of target grating emission angles to corresponding optimal upper-layer tooth lengths, lower-layer tooth lengths, *x*-dimension layer offsets, and period lengths, and, then, we interpolate over this discrete dataset to select an ideal combination of grating parameters for each period within the grating. To generate the discrete dataset, we first define the range of emission angles necessary to enable focusing in the propagation dimension. Then, we perform an iterative parameter optimization for eleven discrete angles within this range using the particle-swarm algorithm. For each of the eleven target angles, we initialize the particle-swarm algorithm in MATLAB^41^ with an initial set of thirteen grating geometries, each defined by three geometric parameters: upper-layer tooth length, lower-layer tooth length, and layer offset in the *x* dimension (all defined as a fraction of the local grating period). Using these geometric parameters, we solve for the length of the grating period analytically, based on the target angle of emission and the effective refractive index of the trial grating^42^. For each of the resulting trial grating geometries, referred to as “particles”, the particle-swarm algorithm calls a Lumerical-FDTD electromagnetic-simulation script^43^ that constructs a non-apodized grating with the given geometric parameters (setting the same upper-layer tooth length, lower-layer tooth length, *x*-dimension layer offset, and period length for all periods in the grating). We then simulate the performance of this grating with a given input polarization (TE or TM). Once the simulation is complete, we integrate the beam profile recorded above the grating to determine the fraction of the optical power, $P_{ion}$, emitted in a small range around the desired target angle. Next, we compute $1 - P_{ion} - P_{rem}$, where $P_{rem}$ is the fraction of the optical power remaining at the end of the simulated grating due to incomplete scattering, and feed the value of $P_{ion}/(1 - P_{ion} - P_{rem})$ back into the MATLAB algorithm as a figure of merit representing the ratio of desired-angle to undesired-angle scattering. Based on the figures of merit calculated for each particle, the particle-swarm algorithm then assigns a new set of grating-geometry parameters to each particle, bounded by a minimum-feature-size constraint. This parameter-selection, Lumerical-simulation, and figure-of-merit-calculation process repeats until the figure of merit is maximized, indicating an optimal parameter combination has been reached. We complete this parameter optimization for each of the eleven discrete target emission angles within our defined angle range, resulting in a dataset that maps each target emission angle to a corresponding optimal grating geometry (i.e. optimal upper-layer tooth length, lower-layer tooth length, *x*-dimension layer offset, and period length), as plotted in Fig. 3d, e. Figure 3f, g shows the resulting fraction of power emitted towards the desired ion location and the resulting emission angle, respectively, for each of the optimized grating geometries in the dataset, confirming the desired unidirectionality and directivity. Finally, we utilize this dataset to construct the final apodized grating in a period-wise fashion. For each of the approximately forty periods within the grating, we calculate the necessary local emission angle based on the *x* position of the grating period, the fixed *x* position of the target ion location, the fixed *z* position of the target ion location, and the refraction of the emitted light at the cladding-to-air interface (Fig. 2a). Then, we interpolate over the discrete dataset to select the optimal upper-layer tooth length, lower-layer tooth length, *x*-dimension layer offset, and period length corresponding to the necessary local emission angle. We repeat this interpolation and selection procedure for each period in the grating to generate the final apodized grating.

Following this parameter-selection process, we simulate free expansion of the input waveguide mode within a slab to determine the phase front of the modes propagating into the grating. This allows us to determine the optimal curvature for each grating tooth to allow for lateral-dimension focusing^44^. Finally, we run a full-scale 3D-FDTD simulation of the designed grating and verify that it performs as intended.

We perform this grating-design process for gratings of multiple different lengths in order to converge on a pair of optimized TE and TM gratings with matching beam intensities at the target ion location, a necessary condition for many ion operations, including the generation of high-purity circular polarization within a TE-TM polarization gradient^25^. Optimizing the grating length is necessary to perform intensity matching between TE and TM gratings due to the fundamentally different scattering behaviors of TE- and TM-polarized modes; as TE waveguide modes are more highly confined than TM modes and, therefore, have higher effective indices of refraction^45^, TE-polarized light scatters more strongly than TM-polarized light from grating emitters with a given minimum feature size. For this reason, the maximum effective aperture size of a fabricable TE grating is intrinsically shorter than that of a TM grating, resulting in fundamentally-improved beam-focusing abilities for TM gratings. Thus, an optimal grating length will truncate the effective aperture of the TM grating such that it focuses with an intensity comparable to the TE grating at the ion location. To determine this optimal grating-aperture size, we design multiple TE-TM grating pairs with different lengths and compare the intensities of the gratings in each pair. After completing this process, we choose a final aperture size that results in matched intensities, with a final result of 17 $\times$ 18 µm in this particular case. The simulated efficiencies of the resulting intensity-matched TE and TM gratings, defined as the percentage of light concentrated in a small (2 × 2 μm) region surrounding the target ion location, are 18.4% and 18.8%, respectively.

To compensate for potential fabrication-bias-induced deviations in grating performance from simulation, we then design a suite of grating variants. First, we pick a nominal target ion location around which we center our variants. We choose an ion location of *x* = 70.7 μm from the start of the 10-μm-long grating-input tapers at a height of *z* = 50 μm, with our ion height fixed by our radio-frequency trap geometry^32^. For both the TE and TM polarizations, we then design seven 17-μm-long gratings each targeting a slightly different *x*-axis position (61.7 to 79.7 μm, in steps of 3 μm), with all variants assuming a constant *z* = 50 μm ion height. In addition, to compensate for potential fabrication-bias-induced deviations in scattering strength and therefore effective-aperture sizes, we design grating variants with multiple lengths. Specifically, for both the TE and TM polarizations, we design three 15-μm-long gratings (targeting positions of *x* = 62.7 μm, 68.7 μm, and 74.7 μm) and three 19-μm-long gratings (targeting positions of *x* = 66.7 μm, 72.7 μm, and 78.7 μm), with all variants assuming a constant *z* = 50 μm ion height.

**Fig. 4:**
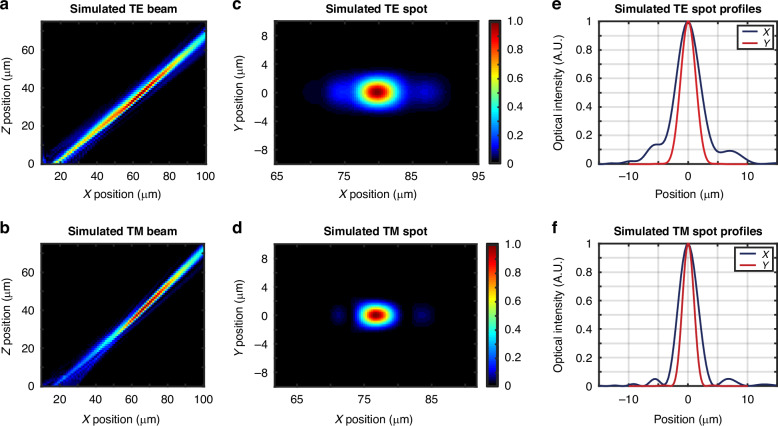
Polarization-diverse integrated-photonics grating-emitter simulated performance. Simulated *xz-*plane beam-intensity profiles for an example characteristic (**a**) TE grating (17 μm long and targeting *x* = 79.7 μm) and (**b**) TM grating (17 μm long and targeting *x* = 76.7 μm). Simulated spots in the *xy* plane at *z* = 50 μm for the characteristic (**c**) TE and (**d**) TM grating. Simulated spot profiles in the *x* (blue) and *y* (red) dimension at *z* = 50 μm for the characteristic (**e**) TE and (**f**) TM grating

The resulting simulated emission profiles in the *xz* plane for an example pair of characteristic gratings are plotted in Fig. 4a, b. The example TE grating (17 μm long and targeting *x* = 79.7 μm) has an expected emission angle of 50.3° and a spot size (1/e^2^ diameter) of 9.9 μm × 5.0 μm at the ion location 50 μm above the chip surface, as shown in Fig. 4c, e. The example TM grating (17 μm long and targeting *x* = 76.7 μm) has an expected emission angle of 46.9° and a spot size of 6.1 μm × 3.7 μm at the ion location 50 μm above the chip surface, as shown in Fig. 4d, f.

### Experimental demonstration of polarization-diverse grating-emitter pairs

After completing the grating-design process, we fabricate and optically test our suite of 13 TE and 13 TM gratings to verify their compatibility with our polarization-gradient-cooling architectures.

We fabricate our gratings in a 200-mm wafer-scale fabrication process developed at MIT Lincoln Laboratory for low-loss routing at wavelengths spanning the spectrum from ultraviolet to near infrared^16^. The platform (Fig. 2a) contains three 100-nm-thick waveguiding layers: one in amorphous aluminum oxide (Al_2_O_3_) and two in silicon nitride (SiN), each separated by 90 nm of silicon dioxide (SiO_2_). The waveguiding layers are clad in 5 μm of SiO_2_, followed by a niobium (Nb) surface-electrode layer. (See Materials and Methods for details.)

**Fig. 5:**
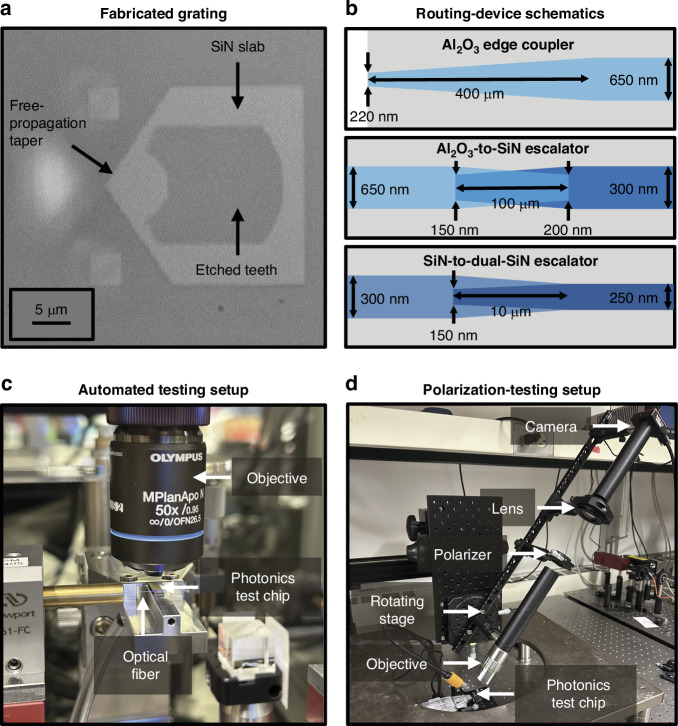
Measurement setups and waveguide-layer routing devices. **a** Top-view micrograph of a fabricated TE grating. **b** Simplified top-view schematics (not to scale) of the integrated-photonics waveguide-based inverse-taper fiber-to-chip edge coupler (top), Al_2_O_3_-to-SiN layer-transition escalator (center), and SiN-to-dual-SiN layer-transition escalator (bottom). **c** Photograph of the automated testing setup used to experimentally characterize the gratings and systems developed in this work, depicting an input optical fiber coupled to a photonics test chip mounted on a vacuum chuck underneath a 50x objective. **d** Photograph of the testing setup used to experimentally characterize the polarization of the gratings developed in this work, depicting a photonics test chip imaged by a polarization-filtering optical system on a rotating platform

To characterize the fabricated gratings (Fig. 5a), we route light from a benchtop laser to the photonics test chip through a series of polarization-maintaining fibers. To set the polarization at the input of the chip, we fix a polarizing beamsplitter cube to a mount aligned with the chip axes. Then, we use transmission through the beamsplitter ports to align the incident polarization of the fiber to the operating polarization of the grating under test (either TE or TM). We then remove the cube and couple light onto the chip using an on-chip Al_2_O_3_ inverse-taper fiber-to-chip edge coupler (Fig. 5b). The light in the Al_2_O_3_ waveguide is then routed through a series of integrated layer-transition escalators: the first transitioning from Al_2_O_3_ to the first SiN waveguiding layer (Fig. 5b) and the second from SiN to dual-layer SiN (Fig. 5b)^16^. Finally, the light is routed to one of the dual-layer grating emitters.

**Fig. 6:**
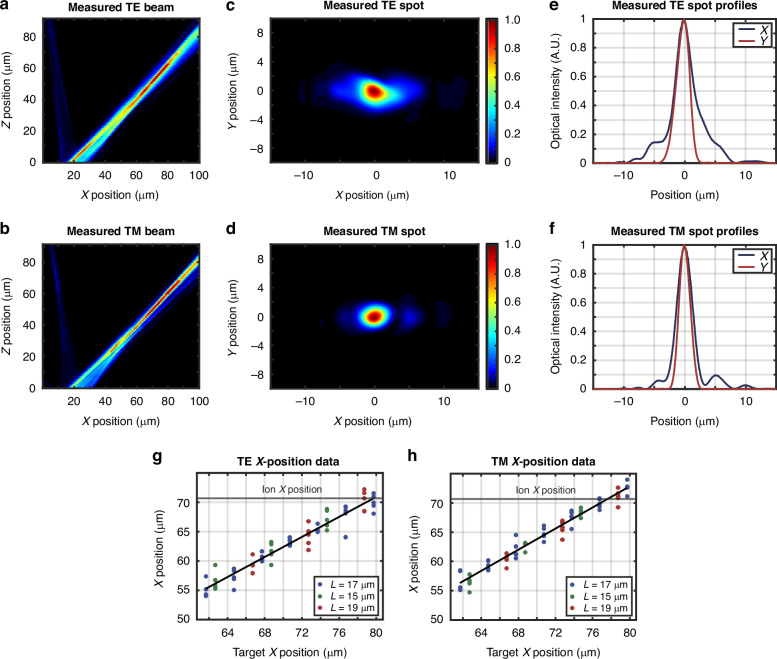
Polarization-diverse integrated-photonics grating-emitter experimental results. Experimentally-measured *xz-*plane beam-intensity profiles for a characteristic (**a**) TE grating (17 μm long and targeting *x* = 79.7 μm) and (**b**) TM grating (17 μm long and targeting *x* = 76.7 μm). Experimentally-measured spots in the *xy* plane at *z* = 50 μm for the characteristic (**c**) TE and (**d**) TM grating. Experimentally-measured spot profiles in the *x* (blue) and *y* (red) dimension at *z* = 50 μm for the characteristic (**e**) TE and (**f**) TM grating. Experimentally-measured versus target beam *x* positions at *z* = 50 μm for (**g**) 13 TE and (**h**) 13 TM grating variants from 5 chips with grating lengths of 17 μm (blue), 15 μm (green), and 19 μm (red) (total of 130 gratings measured)

We image the grating emission using a 50x objective and a visible-light camera. To measure each grating’s performance, we use an automated setup (Fig. 5c) to increment the height of the imaging optical train in 1-μm steps and capture images of the grating’s emitted beam over a range of 0 to 100 μm above the surface of the chip (Fig. 6c, d). We then use the resulting data to compute the grating’s emission angle and beam dimensions (Fig. 6a, b, e, f). Using this characterization procedure, we find that a characteristic fabricated TE grating (17 μm long and targeting *x* = 79.7 μm) successfully emits TE-polarized light at an angle of approximately 43.2° (Fig. 6a) with a spot size (1/e^2^ diameter) of 7.6 μm × 4.3 μm at 50 μm above the chip surface (Fig. 6c). Similarly, a characteristic fabricated TM grating (17 μm long and targeting *x* = 76.7 μm) successfully emits TM-polarized light at an angle of approximately 43.1° (Fig. 6b) with a spot size of 5.0 μm × 3.6 μm at 50 μm above the chip surface (Fig. 6d).

As the optimal ion height (*z* = 50 μm) is a fixed property of the chips’ radio-frequency trapping potential, it is necessary to determine the offset of the emitted beams from a target ion *x* position at this height. Using the characterization procedure introduced above, we image the beams emitted by each of the 26 grating variants within the intended ion-height plane at *z* = 50 μm. Within this plane, we determine the *x* position of the emitted beam and calculate the offset from the target design value. We collect this *x*-position data for all 26 grating variants from five chips sampled from disparate locations across two fabricated wafers (total of 130 gratings measured), to provide a robust analysis of grating performance across sites subject to differing fabrication conditions. The results, plotted in Fig. 6g, h, demonstrate that grating performance remains highly consistent across different chips and wafers. Although consistent, we observe a fabrication-induced bias in our measured *x* positions. While our nominal TE and TM gratings were both originally designed to target *x* = 70.7 μm, the fabricated nominal TE and TM gratings have measured *x* positions of approximately 63.2 $\pm$ 0.6 μm and 64.9 $\pm$ 1.1 μm, respectively. We expect that this discrepancy is due to fabrication bias reducing the effective index of the modes propagating in the gratings. To confirm this hypothesis, we perform scanning-electron-microscope (SEM) cross-sectional imaging of gratings from four chips spanning multiple regions of the two fabricated wafers. From these images, we observe that several factors indeed contribute to a reduction of the effective indices of the gratings. In particular, we find that, on average, the SiN waveguiding layers are biased thinner than the nominal 100 nm, the SiO_2_ gaps between the SiN waveguiding layers are biased thicker than the nominal 90 nm, and the SiN teeth are etched shorter than nominally designed. To verify that this fabrication bias accounts for the observed discrepancy between the simulated and measured grating performance, we modify our 3D-FDTD grating simulations to account for these three bias factors and confirm that they account for the majority of the deviation between the simulated and measured beam *x* positions. Since this observed fabrication bias is highly repeatable across all 130 measured gratings, we anticipate that we can reliably compensate for these grating-emission-angle deviations in future ion-trap grating configurations by using alternate grating variants that exhibit measured *x* positions within close range of the target *x* position of 70.7 μm. For example, to target this position of *x* = 70.7 μm, we can select the TE grating originally designed to target *x* = 79.7 μm and the TM grating originally designed to target *x* = 76.7 μm, as shown in Fig. 6g, h.

Next, we experimentally characterize the efficiencies of the fabricated gratings. To perform this characterization, we first determine the conversion from raw image-brightness values to optical power for the grating-imaging camera. To do this, we couple light from a 422-nm-wavelength laser diode into an optical fiber and align the fiber’s output facet with our visible-light camera. Then, we capture images of the fiber facet at 25 different output powers, as recorded by a power meter, sum the reported pixel-brightness values of the facet images, and fit a line mapping the integrated image brightness values to optical power. Next, to isolate the grating efficiencies from other on-chip loss mechanisms, we must account for losses in the waveguide routing. By repeatedly experimentally measuring fiber-to-chip coupling losses, Al_2_O_3_-to-SiN escalator losses, and SiN-to-dual-SiN escalator losses across multiple chips and wafers, we find an expected total average routing loss of approximately 9.7 dB for TE and approximately 9.3 dB for TM polarization, which we account for in our reported grating efficiencies. Finally, we experimentally measure the power emitted from all 26 grating variants from five chips (total of 130 gratings measured) and calculate their final grating efficiencies, defined as the percentage of light concentrated in a small (2 × 2 μm) region surrounding the beam centroid at *z* = 50 μm. We find that the efficiencies span approximately 5–15% across the different variants, closely matching our simulated efficiency values.

Finally, we experimentally characterize the polarization of the emitted beams. To perform this characterization, we use an infinity-corrected imaging system with a wire-grid polarizer positioned between the objective and the tube lens, as shown in Fig. 5d. Similar to^46^, the imaging system is positioned on a rotating stage, which allows us to orient the axis of the imaging system along the emission angle of a given beam. To characterize the polarization of each grating, we set the polarization at the input of the chip to the operating polarization of the grating (either TE or TM) and image the emitted beam for two orthogonal polarizer configurations, one aligned to admit only light polarized along the *y* axis of the grating (*y*-polarized light, corresponding to TE polarization) and the other aligned to admit only light polarized orthogonal to the *y* axis (*xz*-polarized light, corresponding to TM polarization), using the coordinate basis defined in Fig. 4a–d. Using this measurement technique, we observe that the polarization of the emitted beams is neither strictly *y*-polarized nor *xz*-polarized, as expected due to the quasi-polarized nature of the waveguide modes supported by the gratings. As physical waveguide modes are bounded in three dimensions, they do not exhibit pure TE or TM polarization; instead, waveguide modes exhibit strong TE- or TM-like polarization, with non-zero electric-field contributions along all three principal axes^42^. As a result, beams emitted by waveguide-based devices also generally exhibit multiple non-zero electric-field components with differing amplitude distributions; we expect a TE grating to emit a beam that is purely *y*-polarized at the center with two lower-power off-center lobes of *xz*-polarized light^36^, whereas we expect a TM grating to emit a beam that is purely *xz*-polarized at the center with two lower-power off-center lobes of *y*-polarized light. Due to the spatial variation of the *y*- and *xz*-polarized components of these beams, it is of interest to characterize both the polarization purity at the center of the beams, where we expect a pure *y* or a pure *xz* polarization, as well as the total power contained in each of the two polarization components. First, we characterize the polarization purity at the center of the beams emitted by a characteristic TE grating and a characteristic TM grating. For the TE grating, we measure an extinction ratio of $> \, 15 \, {\rm{dB}}$ for the *xz*-polarized component, with the measurement bounded by the noise floor of our microscope camera. Similarly, for the TM grating, we measure an extinction ratio of $> \, 15 \, {\rm{dB}}$ for the *y*-polarized component, likewise bounded by the camera noise floor. Accordingly, both gratings exhibit a polarization purity of > 97% at the center of their respective beams, in strong agreement with our expectations. Second, for each grating, we integrate the total power in the *y*- and *xz*-polarized components of the emitted beam ($P_{y}$ and $P_{xz}$, respectively), with the total power in the beam given by ${P}_{{total}}={P}_{y}+{P}_{{xz}}$. From our grating simulations, we expect TE gratings to exhibit a ratio of $P_{y}/P_{total}$ $\approx$ 0.95 ($P_{xz}/P_{total}$ $\approx$ 0.05) and TM gratings to exhibit a ratio of $P_{xz}/P_{total}$ $\approx$ 0.995 ($P_{y}/P_{total}$ $\approx$ 0.005). For the TE grating, we experimentally measure a ratio of $P_{y}/P_{total}$ $\approx$ 0.95 ($P_{xz}/P_{total}$ $\approx$ 0.05), in strong agreement with the simulated ratio. For the TM grating, we experimentally measure a ratio of $P_{xz}/P_{total}$ $>$ 0.99 ($P_{y}/P_{total}$ $<$ 0.01), likewise in strong agreement with the simulated ratio (with the TM-grating measurement again bounded by the noise floor of the camera).

### Experimental demonstration of integrated-photonics-based systems for polarization-gradient cooling

After characterizing our individual TE and TM gratings, we utilize them as building blocks to experimentally demonstrate our three proposed polarization-gradient-cooling systems.

We implement two different system categories – single- and dual-fiber-input systems – to enable multiple modalities of polarization-gradient cooling. The dual-fiber-input systems with splitting performed off chip are subject to phase instability induced by vibrations and drift in the off-chip splitter and off-chip dual-fiber routing. Therefore, the dual-fiber-input systems are conducive to traveling-wave cooling implementations similar to prior free-space polarization-gradient-cooling demonstrations^24,27,28^. Alternatively, the single-fiber-input systems with splitting and routing performed on chip benefit from the intrinsic relative path-length stability offered by on-chip functionality and are therefore capable of producing phase-stable polarization gradients (as quantified in the trapped-ion-result section below). Consequently, the single-fiber-input systems enable cooling at specific phases within this stable gradient for the first time, as demonstrated in the following section, illustrating the unique advantage afforded by integrated-photonics-based systems.

**Fig. 7:**
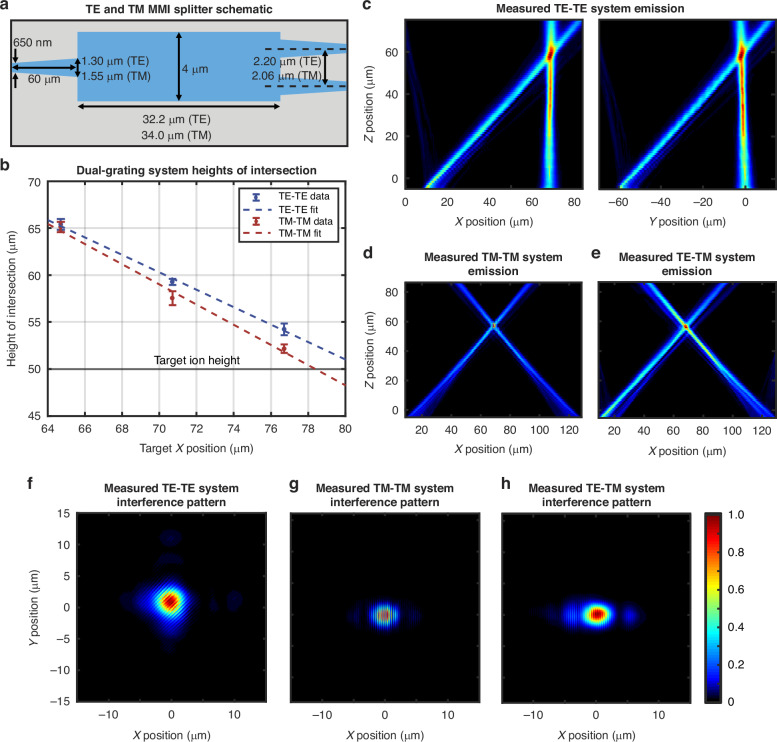
Integrated-photonics-based polarization-gradient-cooling systems experimental results. **a** Simplified schematic (not to scale) of the TE and the TM integrated-photonics Al_2_O_3_ multimode-interferometer (MMI) splitter with relevant dimensions labeled for each polarization. **b** Measured beam-intersection heights for the TE-TE (blue) and TM-TM (red) systems compared to the target ion height of *z* = 50 μm (black) (total of 48 systems measured). **c** Experimentally-measured *xz*-plane (left) and *yz*-plane (right) emission profiles for a TE-TE system containing two nominal TE gratings. Experimentally-measured *xz*-plane emission profiles for (**d**) a TM-TM and (**e**) a TE-TM system. Experimentally-measured *xy*-plane views of the emission from the (**f**) TE-TE system at *z* = 60 μm, (**g**) TM-TM system at *z* = 59 μm, and (**h**) TE-TM system at *z* = 58 μm, showing the resulting dual-beam interference patterns

We begin by introducing and characterizing the single-fiber-input (TE-TE and TM-TM) systems. These systems consist of an on-chip inverse-taper Al_2_O_3_ edge coupler routed to a polarization-specific (TE- or TM-optimized) on-chip Al_2_O_3_ multimode-interferometer (MMI) splitter that splits light from a single input Al_2_O_3_ waveguide into two output Al_2_O_3_ waveguides (Fig. 7a)^47^. These separate waveguides are then routed through the integrated Al_2_O_3_-to-SiN and SiN-to-dual-SiN escalators discussed in the previous section. Finally, each dual-layer arm routes to a single integrated grating emitter, with the grating orientation determined by the desired beam configuration. The gratings are oriented orthogonally in the TE-TE case and opposite each other in the TM-TM case, as shown in Fig. 2b, c. We fabricate these systems using the same 200-mm wafer-scale process introduced in the previous section and depicted in Fig. 2a^16^.

To test these systems, we utilize the same setup used in the previous section, shown in Fig. 5c. We once again image the chip with a 50x objective and a visible-light camera, set our input polarization using a free-space polarizing beamsplitter, and use our automated setup to increment the height of the imaging optical train in 1-μm steps, this time capturing the light emitted by both gratings simultaneously. Characteristic side-view and top-down scan results for the TE-TE and TM-TM systems are provided in Fig. 7c, d, f, g.

To characterize the effects of the fabrication bias on these systems, we measure the performance of three TE-TE- and three TM-TM-system variants. Each system variant contains a distinct pair of TE- or TM-grating variants. Each grating-variant pair targets a different *x* position, corresponding to a different angle of emission and, accordingly, a different height of intersection, *z*, for the two-grating systems. Specifically, we target *x* = 64.7 μm, 70.7 μm, and 76.7 μm. By measuring the performance of each of these variants, we can characterize the fabrication-bias-induced deviation of our measured intersection heights from the target design values. We measure these system variants across 8 chips from 3 wafers (total of 48 systems measured) and fit trendlines to the data (Fig. 7b). We find that our linear fits strongly agree with the data presented in the previous section for our single-grating test-structure *x* positions; to target a beam-intersection height of *z* = 50 μm (corresponding to *x* = 70.7 μm), we can use the TE grating originally designed to target *x* = 79.7 μm and the TM grating originally designed to target *x* = 76.7 μm.

From the top-down views of the single-fiber-input systems, we find that the gratings emit with closely-matched intensities. This is due to the even splitting ratios of the integrated MMI devices; for the TE-optimized and TM-optimized MMIs, we measure splitting ratios of −3.02$\pm$0.36 dB and −2.93$\pm$0.24 dB, respectively, with the target -3.01 dB ratio within a single standard deviation for both MMIs.

Notably, at the intersection height of each beam pair (Fig. 7f, g), we observe an optical-intensity gradient along the polarization-gradient axis. While theory would predict that beams with perfectly orthogonal polarizations will not generate an observable interference pattern, we find that these patterns are due to both the alignment of the magnetic fields of our beams as well as the quasi-TE / quasi-TM nature of waveguide modes. As detailed in the grating-characterization section above, the beams emitted by the TE and TM gratings exhibit both *y*-polarized and *xz*-polarized electric-field components, which contribute to the observed interference patterns. However, these contributions alone do not dictate the interference pattern of these systems. For example, for the particular case of the TM-TM systems, the beams are aligned such that their magnetic fields exhibit a near-total overlap. In contrast, the TE-TE systems are aligned such that the dual-beam magnetic-field overlap is significantly reduced. This results in a larger magnetic-field interference contribution for the TM-TM systems than for the TE-TE systems. The final interference patterns generated by each dual-grating system are thus a result of several factors including the angle between the beams, the polarization purity of the emitted waveguide modes, and the angle of emission. Despite the hybrid-polarization emission and intensity interference patterns, we successfully observe cooling, as demonstrated in the following section. We expect that this polarization impurity decreases the purity of our polarization gradient’s circular-polarization regions, leading to a decreased difference in the *g*_±1/2_ light shift and, therefore, reduced cooling efficiency. Further parametrizing our dual-grating-system interference patterns and quantifying the effects of interference on our polarization gradient could be explored further in future work.

After thoroughly characterizing the single-fiber-input polarization-gradient-cooling systems, we expand upon our testing setup to also characterize dual-fiber-input systems. Our dual-fiber-input structures begin with one on-chip inverse-taper Al_2_O_3_ edge coupler on either side of the photonics test chip. Then, each separate Al_2_O_3_ waveguide is routed through the same integrated Al_2_O_3_-to-SiN and SiN-to-dual-SiN escalators discussed above. Finally, each arm terminates in a single grating: one TE- and the other TM-emitting, as shown in Fig. 2d. To test these systems, we route light from a benchtop laser to a single-mode fiber routed through polarization paddles. We then couple this single-mode fiber to a fiber-based polarizing beamsplitter with a single-mode-fiber input and two polarization-maintaining-fiber outputs. Finally, we align one of the two polarization-maintaining-fiber arms to each side of the photonics test chip, set the polarization at the respective inputs to TE and TM using a polarizing beamsplitter cube, and couple light onto the chip using the two on-chip Al_2_O_3_ inverse-taper edge couplers.

Characteristic measurement results for the dual-fiber-input systems are provided in Fig. 7e, h. While we demonstrate that these dual-fiber-input systems remain sufficiently stable for us to image an interference pattern within the exposure time of the camera (approximately 100 μs), these dual-fiber-input systems are inherently less stable than the single-fiber-input systems demonstrated above, due to increased susceptibility to relative phase variability between the two paths induced by using an off-chip fiber-based, rather than an on-chip, splitter. As a result, the dual-fiber-input systems are better suited for polarization-gradient cooling implementations with a traveling-wave gradient, more similar to previous free-space demonstrations^24,27,28^. To instead achieve the long-term stability offered by single-fiber-input systems, we could implement our TE-TM architecture with an integrated-photonics polarization rotator operating at 422 nm, as we developed and experimentally demonstrated in^48^. Such an architecture would allow us to couple TE-polarized light onto the chip using a single input edge coupler, route this light to an on-chip TE-optimized MMI, route one of the MMI-output arms through a polarization rotator to convert the arm’s polarization from TE to TM, then route each arm to a polarization-specific grating, as we outlined in^49^.

### Experimental demonstration of polarization-gradient cooling of a trapped ion

Using the single-fiber-input TE-TE polarization-gradient-cooling system discussed and optically characterized above, we demonstrate integrated-photonics-based polarization-gradient cooling of a trapped ion for the first time.

For this demonstration, we utilize a planar electronic-photonic trap chip containing a trap site with one of the single-fiber-input TE-TE systems. This system contains two of the nominal TE gratings, designed to form beams with an intersection point 50 μm over the surface of the trap chip. We install this trap chip in a cryogenic vacuum system^50,51^ for the purpose of achieving ultrahigh vacuum through cryopumping. The cryogenic environment is equipped with a fiber-feedthrough system to allow for coupling of light onto the chip from an off-chip 422-nm-wavelength laser located outside of the cryogenic system.

**Fig. 8:**
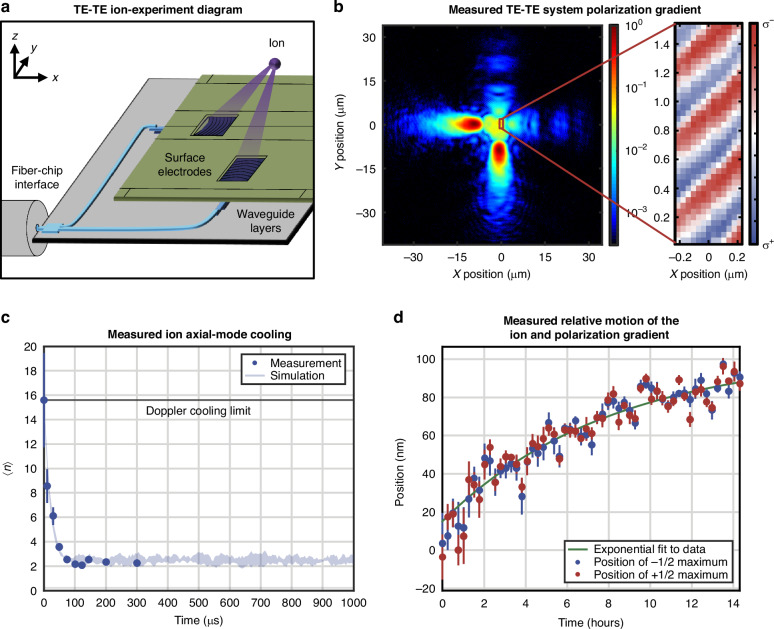
Integrated-photonics-based polarization-gradient-cooling ion results. **a** Conceptual diagram of integrated-photonics-based polarization-gradient cooling of a trapped ion, depicting light coupled onto a chip from an optical fiber, routed through integrated-photonics waveguiding layers, and emitted upward towards a common ion location through surface-electrode windows (not to scale). **b** Experimentally-measured *xy*-plane view at *z* = 50 μm of the emission from the nominal TE-TE system (in log scale), with an inset depicting the resulting polarization gradient, characterized using fluorescence from a trapped ^88^Sr^+^ ion (data also included in^32^). **c** Experimentally-measured (circles) and simulated (line) average motional state (〈*n*〉) of the 1.2-MHz axial mode versus time for a ^88^Sr^+^ ion in the generated polarization gradient (data also included in^32^), demonstrating successful polarization-gradient cooling to significantly below the Doppler cooling limit. **d** Experimentally-measured position versus time of the *g*_1/2_ population maximum (red) and *g*_-1/2_ population maximum (blue) for a ^88^Sr^+^ ion in the generated polarization gradient, demonstrating maximal motion of approximately 10 nm/hour and confirming the passive phase stability of the integrated-photonics-based polarization gradient

Once the electronic-photonic trap chip is installed, we load ^88^Sr^+^ ions by photoionizing neutral Sr atoms from a remote precooled source^50,51^. Upon loading an ion, we shuttle the ion to a polarization-gradient-cooling zone located approximately 240 μm away along the axial dimension of the trap chip. This zone contains the two orthogonally-oriented TE gratings comprising our single-fiber-input TE-TE polarization-gradient-cooling system, as depicted in Fig. 8a.

As characterized above, the fabricated gratings in our polarization-gradient-cooling system exhibit an offset from their designed height of intersection, causing the highest-intensity regions of the emitted beams to intersect approximately 9 μm higher than the target height of 50 μm. As this intersection is far from the optimal operating range around the null of the trap chip’s integrated radio-frequency Paul trap, ions positioned at the dual-beam intersection are not well confined. Therefore, we confine ions within the trap null at a height of 50 μm and implement polarization-gradient cooling at the intersection of lower-intensity portions of the beams, as depicted in Fig. 8b.

Once the ion reaches its target position, we power the gratings in the polarization-gradient-cooling system and observe a unique feature of the resulting polarization gradient: the interferometric stability offered by the single-fiber-input system with splitting performed on chip allows for the formation of a phase-stable polarization gradient, akin to previously demonstrated integrated-photonics-based phase-stable standing waves^52^. In this section, we present a brief summary of the parameter optimization and cooling performance attained using the inherent phase stability of this system. We present a more thorough contextualization, discussion, and demonstration of ion dynamics within this phase-stable gradient in an accompanying publication^32^ (whereas the focus of this publication is on the conceptualization, design, experimental demonstration, and rigorous characterization of the devices and systems used to enable integrated-photonics-based polarization-gradient cooling).

The passive phase stability of our system facilitates several functionalities, including spatial mapping of the polarization gradient, as depicted in Fig. 8b (experiment further discussed in^32^). While similar measurements have previously been demonstrated, they have relied on techniques such as stroboscopic sampling to map a running-wave gradient^53^. To map the polarization gradient of our system, we use a measurement scheme based on selective state preparation. In summary, we apply a polarization-gradient pulse, resulting in the spatially-dependent preparation of the ion in one of the ground-state Zeeman sublevels. Then, we selectively excite the ion along the ${\rm{S}}_{1/2}\, \to \, {\rm{D}}_{5/2}$ transition and use the ion’s fluorescence to evaluate the sublevel occupation (a process often referred to as electron shelving^54^). Based on the population in each Zeeman sublevel, we determine the local polarization. Despite the fabrication-induced offset in the height of intersection of our beams, we find that our polarization gradient closely matches the theoretically-anticipated gradient calculated in^32^. In particular, we measure a polarization gradient along $\hat{x}-\hat{y}$ with an effective gradient wavelength, ${\lambda }_{{eff}}$, of 555 nm (in comparison with an anticipated ${\lambda }_{{eff}}$ of 597 nm, derived from the target angle between the two beams and the angle of the polarization gradient with respect to the beams). Moreover, we observe an upper bound on the population fringes of 70%, primarily limited by the thermal state of the ion’s motion, as analyzed in^32^, with an additional contribution from the presence of undesired $\pi$-polarized light (a contribution of approximately 7–13%, believed to be a result of the presence of the non-orthogonal components of the quasi-polarized beams discussed in the previous section).

Beyond facilitating polarization-gradient mapping, the phase stability of our system allows us to choose a specific phase of the polarization gradient and thoroughly study the cooling / heating dynamics over a range of system parameters. In Fig. 8c (experiment further discussed in^32^), we plot the time dynamics of the cooling process in this phase-stable polarization gradient for one parameter set. Specifically, we plot the average motional state (〈*n*〉) of the 1.2-MHz axial mode versus time for a trapped ^88^Sr^+^ ion initialized with Doppler cooling. The decay of the motion to 〈*n*〉 = 2.33 $\pm$ 0.18 demonstrates successful polarization-gradient cooling to significantly below the Doppler limit (〈*n*〉 ≈ 10), in close agreement with numerical simulations^32^. We observe cooling to near the asymptotic limit within around 100 ${\rm{\mu }}{\rm{s}}$, indicating a cooling-speed advantage in comparison with resolved-sideband cooling, which can require on the order of 1 ms to complete^23^. In^32^, we demonstrate further results corresponding to parameter sweeps over ion position, frequency detuning, and optical power and compare the results to theoretical models, demonstrating strong agreement between measured and predicted ion-cooling performance.

Finally, in this publication, we characterize the stability of our ion / polarization-gradient system by measuring the relative position between the ion and polarization gradient over the course of approximately 15 hours. At each point in time, we fit a sine function to both the *g*_-1/2_ and *g*_1/2_ population data collected in state-preparation experiments while scanning the ion position along the trap axis through the polarization gradient. We then convert the relative phase of the sine functions to a positional translation of the ion relative to the polarization gradient. Using this process, we measure a maximum relative drift of approximately 10 nm/hour immediately following charging of the electronic-photonic trap-chip surface induced during ion loading. As the trap surface discharges, the displacement rate of the polarization gradient exponentially decays, as depicted in Fig. 8d. Given that the relative motion between the ion and the polarization gradient diminishes over time, we anticipate that the ion and polarization gradient will remain increasingly stable over longer timeframes. Furthermore, to compensate for this small relative displacement between the ion and polarization gradient over time, we can use a closed-loop feedback scheme detailed in^32^ to adjust the position of the ion with respect to the polarization gradient. Using this scheme, we further stabilize the relative position between the ion and the polarization gradient to $< 10\,{\rm{nm}}$ (i.e., approximately 0.02${\lambda }_{{eff}}$).

## Discussion

In this paper, we designed and experimentally demonstrated key polarization-diverse integrated-photonics devices and utilized them to implement a variety of integrated-photonics-based polarization-gradient-cooling systems, culminating in the first experimental demonstration of polarization-gradient cooling of a trapped ion by an integrated-photonics-based system.

First, we discussed the mechanisms underlying polarization-gradient cooling and introduced several integrated-photonics-based architectures capable of performing polarization-gradient cooling. Second, we designed and experimentally demonstrated the key integrated-photonics routing and grating-emitter devices required to implement these architectures. Third, we incorporated these devices into, and successfully experimentally demonstrated, multiple integrated-photonics-based polarization-gradient-cooling systems capable of forming either traveling-wave or phase-stable polarization gradients. Finally, we implemented one of the phase-stable systems within an integrated-photonics-based ion-trap chip and used it to demonstrate the first polarization-gradient cooling of a trapped ion using an integrated-photonics-based system.

The demonstrations in this work provide a strong foundation for the further scaling of integrated-photonics-based trapped-ion cooling. By experimentally characterizing 130 gratings and 48 systems sampled across multiple chips and wafers, we have confirmed that the integrated-photonics-based devices and systems presented in this work are highly replicable and can therefore be reliably iterated on for future experiments. Therefore, in the future, we intend to demonstrate polarization-gradient cooling using a second generation of the integrated-photonics-based devices and systems developed in this work, in which we will utilize alternate grating variants to compensate for the fabrication biases characterized in this work. Furthermore, we intend to demonstrate polarization-gradient cooling using more complex integrated-photonics-based systems, including single-fiber-input TE-TM systems, enabled by the incorporation of integrated polarization-control devices demonstrated by our group^48^. In addition, we intend to expand upon the single-ion demonstration reported in this work by extending integrated-photonics-based polarization-gradient cooling to multiple ions, potentially facilitated by the tiling of multiple gratings. Moreover, beyond extending cooling to multiple ions at a single trap site, we also intend to scale our integrated-photonics-based systems to enable polarization-gradient cooling at multiple trap sites, motivated by the field’s progress towards large-scale multi-zone architectures, such as the quantum charge-coupled device^14^. Finally, we intend to explore the co-implementation of integrated-photonics-based Doppler cooling, polarization-gradient cooling, and ground-state cooling techniques such as resolved-sideband cooling to enable highly-efficient ground-state cooling.

More broadly, this work provides a basis for deeper exploration into the application spaces accessible through the formation of phase-stable polarization-diverse structured light fields. Specifically, in the future, we intend to further explore the impact of the quasi-polarized nature of waveguide modes on the polarization purity of beams emitted by integrated gratings and, therefore, on the ion dynamics induced by these beams. In addition, we intend to more rigorously explore the additional degree of freedom introduced by polarization diversity in the formation of structured light fields with varying electric- and magnetic-field interference patterns. Relatedly, we intend to build upon recent progress in the field^29–31^ by designing and experimentally demonstrating additional integrated-photonics-based devices and systems capable of arbitrary polarization emission, building towards future ion demonstrations ranging from polarization-based photon-mediated entanglement^55,56^ to electromagnetically-induced-transparency cooling^49^, and even extending towards enabling polarization-diverse integrated-photonics-based functionalities for neutral atoms^29^.

In conclusion, by demonstrating polarization-gradient cooling using an integrated-photonics-based system, this work paves the way for higher-efficiency integrated-photonics-based trapped-ion computations. Moreover, in general, this work opens up the field of polarization-diverse integrated-photonics-based devices and systems for trapped ions, enabling future advanced operations for integrated-photonics-based trapped-ion platforms. Accordingly, this work has the potential to facilitate further advancements in such platforms that improve upon the functionality of free-space trapped-ion systems with the intrinsic scalability and stability of integrated photonics.

## Materials and Methods

### Integrated-photonics-based device and system fabrication

We fabricate the integrated-photonics-based polarization-gradient-cooling systems and their constituent devices in a 200-mm wafer-scale fabrication process developed at MIT Lincoln Laboratory for low-loss routing at wavelengths spanning the spectrum from ultraviolet to near infrared^16^. The platform (Fig. 2a) contains three waveguiding layers, two metal layers, and one layer of indium tin oxide (ITO).

We begin the fabrication by depositing a 1-μm-thick layer of plasma-enhanced-chemical-vapor-deposition (PECVD) SiO_2_ on a 200-mm silicon wafer. We then deposit a 500-nm-thick sputtered niobium (Nb) metal layer and pattern it via optical lithography to form a ground plane for the ion trap^12^. Next, we deposit a 6.5-μm-thick bottom-cladding PECVD SiO_2_ layer. We anneal the SiO_2_ at 400 °C for 3 hours, and we planarize its top surface using a chemical-mechanical polishing (CMP) step to a thickness of 5.0 µm above the ground metal; this is done both to reduce surface roughness and to remove any step height changes from gaps in the ground metallization. Then, we deposit a 100-nm-thick nitrogen-rich SiN (*n* = 1.895 $\pm$ 0.007 at a wavelength of 633 nm) waveguiding layer using PECVD, pattern the SiN layer using 193-nm deep-UV lithography with an ASML PAS 5500 stepper, and fully etch through the SiN using inductively-coupled-plasma (ICP) reactive-ion etching (RIE) using a SF_6_ etch chemistry. After this etch, we deposit another 1-μm-thick layer of SiO_2_ and perform a CMP step, targeting a final SiO_2_ thickness of 90 nm over the height of the first SiN layer. We then deposit and pattern the second 100-nm-thick SiN layer identically to the first, followed by an additional SiO_2_ layer similar to the first, and, again, CMP this SiO_2_ layer to a thickness of 90 nm above the second SiN waveguide layer. Next, we deposit the third waveguide film, a 100-nm-thick Al_2_O_3_ layer, using atomic layer deposition (ALD), which is used as the primary waveguide routing layer on the chip. We pattern the mask using the same lithography process used previously for the SiN and etch it with a RIE etch using a BCl_3_ etch chemistry. Next, we deposit a 5.7-μm-thick top-cladding layer of PECVD SiO_2_, anneal the SiO_2_ at 400 °C for 3 hours, and perform a CMP step, thinning the SiO_2_ to a final height of about 5.0 µm above the bottom of the second SiN layer or approximately 10.2 µm above the ground metal. Following the final CMP step, we pattern and etch a 10.2-µm-deep ground-connecting via. We then deposit a 1-μm-thick Nb layer, pattern it via optical lithography, and etch it to define the trap electrodes and the windows in the electrodes through which the integrated grating emitters emit their beams (Fig. 2b−d). Then, we deposit and pattern a 50-nm-thick layer of ITO over the surface-electrode windows to mitigate charging of the exposed dielectrics on the trap-chip surface. Finally, we utilize a 16-µm-thick spray-deposited-resist process to define the optical facets for the chip. We first etch through the approximately 12-µm-thick SiO_2_, and then another approximately 175 µm into the silicon substrate to form a deep-etched dicing street, which, after wafer singulation, allow for optical fiber coupling into the Al_2_O_3_ edge couplers that terminate at the chip edges.

## Data Availability

Data are available upon reasonable request.
